# Efficacy of a novel polyoxazoline-based hemostatic patch in liver and spleen surgery

**DOI:** 10.1186/s13017-023-00483-x

**Published:** 2023-03-14

**Authors:** Edwin A. Roozen, Roger M. L. M. Lomme, Nicole U. B. Calon, Richard P. G. ten Broek, Harry van Goor

**Affiliations:** 1 Department of Surgery, RadboudUMC, Geert Grooteplein 10, 6525 GA Nijmegen, the Netherlands; 2 GATT Technologies BV, Nijmegen, the Netherlands

**Keywords:** GATT-patch, Hemostasis, Liver, Patch, Porcine, Sealant, Sealing, Spleen, Surgical bleeding, TachoSil^®^, Veriset™

## Abstract

**Background:**

A new hemostatic sealant based on a *N*-hydroxy-succinimide polyoxazoline (NHS-POx) polymer was evaluated to determine hemostatic efficacy and long-term wound healing and adverse effects in a large animal model of parenchymal organ surgical bleeds.

**Methods:**

Experiment 1 included 20 pigs that were treated with two NHS-POx patch prototypes [a gelatin fibrous carrier (GFC) with NHS-POx and an oxidized regenerated cellulose (ORC) with poly(lactic-co-glycolic acid)-NHS-POx:NU-POx (nucleophilically activated polyoxazoline)], a blank gelatin patch (GFC Blank), TachoSil^®^ and Veriset™ to stop moderate liver and spleen punch bleedings. After various survival periods (1–6 weeks), pigs were re-operated to evaluate patch degradation and parenchymal healing. During the re-operation, experiment 2 was performed: partial liver and spleen resections with severe bleeding, and hemostatic efficacy was evaluated under normal and heparinized conditions of the two previous prototypes and one additional NHS-POx patch. In the third experiment an improved NHS-POx patch (GATT-Patch; GFC-NHS-POx and added 20% as nucleophilically activated polyoxazoline; NU-POx) was compared with TachoSil^®^, Veriset™ and GFC Blank on punch bleedings and partial liver and spleen resections for rapid (10s) hemostatic efficacy.

**Results:**

NHS-POx-based patches showed better (GFC-NHS-POx 83.1%, ORC-PLGA-NHS-POx: NU-POx 98.3%) hemostatic efficacy compared to TachoSil^®^ (25.0%) and GFC Blank (43.3%), and comparable efficacy with Veriset™ (96.7%) on moderate standardized punch bleedings on liver and spleen. All patches demonstrated gradual degradation over 6 weeks with a reduced local inflammation rate and an improved wound healing. For severe bleedings under non-heparinized conditions, hemostasis was achieved in 100% for Veriset™, 40% for TachoSil and 80–100% for the three NHS-POx prototypes; similar differences between patches remained for heparinized conditions. In experiment 3, GATT-Patch, Veriset™, TachoSil and GFC Blank reached hemostasis after 10s in 100%, 42.8%, 7.1% and 14.3%, respectively, and at 3 min in 100%, 100%, 14.3% and 35.7%, respectively, on all liver and spleen punctures and resections.

**Conclusions:**

NHS-POx-based patches, and particularly the GATT-Patch, are fast in achieving effective hemostatic sealing on standardized moderate and severe bleedings without apparent long-term adverse events.

## Background

Intraoperative bleeding can cause significant morbidity and mortality and may have several health economic implications such as a longer operative time, more resources' use and a prolonged hospital stay [1–6]. Rapid control of bleeding is critical, and whenever standard methods such as temporary tamponing, electrocautery and suturing fail, hemostatic products are often used [7–9].

Topical hemostatic agents such as patches, glues, powders and sprays are divided into three categories [10]: (1) adhesive, containing fibrinogen and thrombin [11–13], (2) mechanical, containing gelatin, collagen or oxidized regenerated cellulose (ORC) [14, 15], and (3) sealants containing polyethylene glycol [16, 17]. Current products have several disadvantages regarding indication area, user handling and high costs. Examples of these disadvantages are partial detachment when applied at irregular tissue surfaces, a long waiting time before permanent hemostasis, lower effectiveness in patients with coagulopathy and production from human-derived coagulation components increasing costs [18].

We recently developed a first prototype of a novel hemostatic patch, combining mechanical and sealant properties, that is pliable, holds promise to be fast in stopping a bleeding, can be easily manufactured [19] and has favorable ex vivo degradation and excretion characteristics [20]. The active polymer in the patch consists of a polyoxazoline (POx) base to which multiple *N*-hydroxy-succinimide (NHS) groups are linked. These NHS groups bind to the NH_2_ groups of proteins in human tissues, including blood and proteins on organ surfaces, which causes immediate clotting. This NHS functionalized polyoxazoline-based polymer (NHS-POx) has the potential of being combined with other carriers to form a gel, spray or powder.

Early results of different patch prototypes in a surgical liver injury model in rodents were promising, demonstrating good hemostatic efficacy, with no adverse effects on short-term wound healing [21]. However, blood flow in rodents is relative low and long-term healing and degradation were not assessed. The aim of the current study was to determine hemostatic efficacy in a challenging large animal model of parenchymal organ injury, with a blood flow comparable to that encountered in emergency situations in surgery. Further we assessed long-term wound healing and degradation of NHS-POx patches.

## Methods

### Animals

In total, 28 pigs (Sus scrofa domesticus) were used, 20 for combined experiment 1 and 2, and 8 for experiment 3. The pigs weighed 50–70 kilos and were housed per pairs in a pen with water and standard food ad libitum. A standard 12-h light/dark cycle was maintained. All pigs had a standard acclimatization period of one week before surgery. The pens were enriched with toys. All animals were checked at least twice a day and controls consisted of body weight, temperature, apathetic behavior and poor food intake. Humane endpoints were defined according to the local animal welfare body guidelines. In the event of a deteriorated condition, such as being unable to get up or eating or drinking independently, the veterinarian was contacted to discuss further policy (treatment or withdrawal from the experiment). In between surgeries, animals were housed on a farm after the first postoperative week.

### Hemostatic patches

#### Experiment 1

*Two experimental patches* (1) *GFC-NHS-POx* (GATT Technologies, Nijmegen, the Netherlands): this hemostatic patch consists of a gelatin fibrous carrier (GFC) with NHS-ester functionalized polyoxazoline polymer P(EtOx-c–OH-c-NHS) 60–20-20 (NHS-POx). (2) *ORC-PLGA-NHS-POx* which is an pxidized regenerated cellulose and polylactic-co-glycolic acid (ORC-PLGA Ethicon, New Jersey, USA, 10 × 5 cm) carrier with NHS-POx combined on a 1:1 molar ratio with P(EtOx-NH2) 80–20 (referred to as nucleophilically activated polyoxazoline, NU-POx).

*One negative control* *GFC Blank:* gelatin fibrous carrier (GFC) (Gelita Rapid, origin: porcine, 5 × 7.5 cm, Gelita Medical GmbH, Eberbach, Germany). This patch is a control for the polymer effect.

*Two positive controls* (1) *TachoSil*^®^ (Takeda Austria GmbH, Linz, Austria) is a collagen sponge coated with the human coagulation factors fibrinogen and thrombin [18]. (2) *Veriset™* (Covidien Inc., Mansfield, MA, USA) consists of oxidized regenerated cellulose impregnated with buffer salts, triglycine and a reactive polyethylene glycol [17].

#### Experiment 2

*Three experimental patches* (1) *GFC-NHS-POx 1.5:* GFC with NHS-POx 1.5 is made in the same way as GFC-NHS-POx, but contains 1.5 times more Gelatin and 1.5 times more NHS-POx. (2) *GFC-NHS-POx* and (3) *ORC-PLGA-NHS-POx* (see experiment 1).

*Two positive controls* *TachoSil*^®^ and *Veriset™* (see experiment 1).

#### Experiment 3

*One Experimental patch* *GFC-NHS-POx:NU-POx (*referred to as ‘GATT-Patch’*):* GFC with NHS-POx and added 20% as nucleophilically activated polyoxazoline (NU-POx).

*One Negative control* *GFC Blank* (see experiment 1).

*Two positive controls* *TachoSil*^®^ and *Veriset™* (see experiment 1).

### Study design

The study was approved by the Dutch Animal Ethics Committee (CCD, numberAVD10300 2015 348) and performed in the Central Animal Laboratory of Radboud University. The experiments were performed according to ARRIVE guidelines [22].

**Fig. 1:**
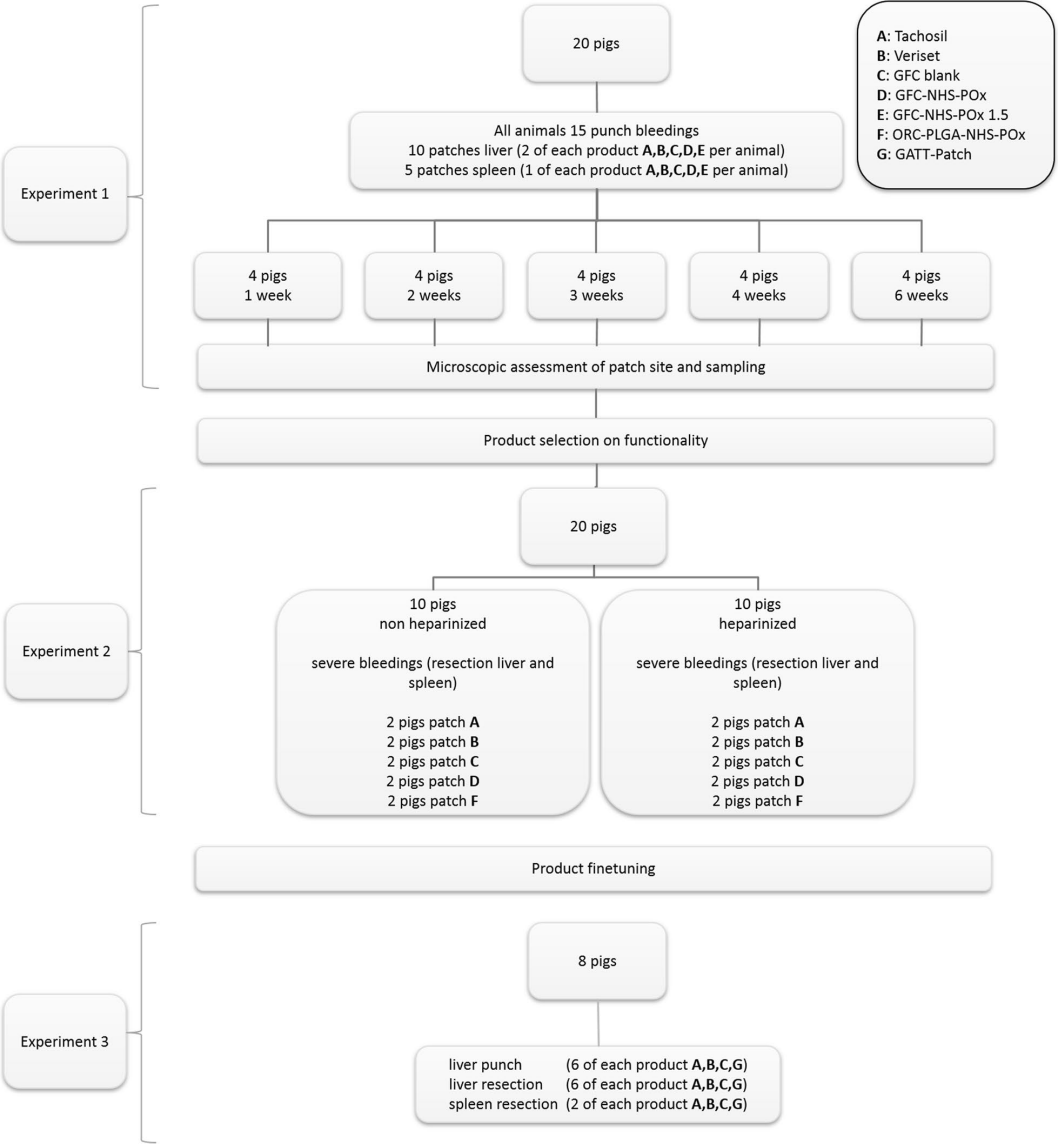
Study design

**Fig. 2:**
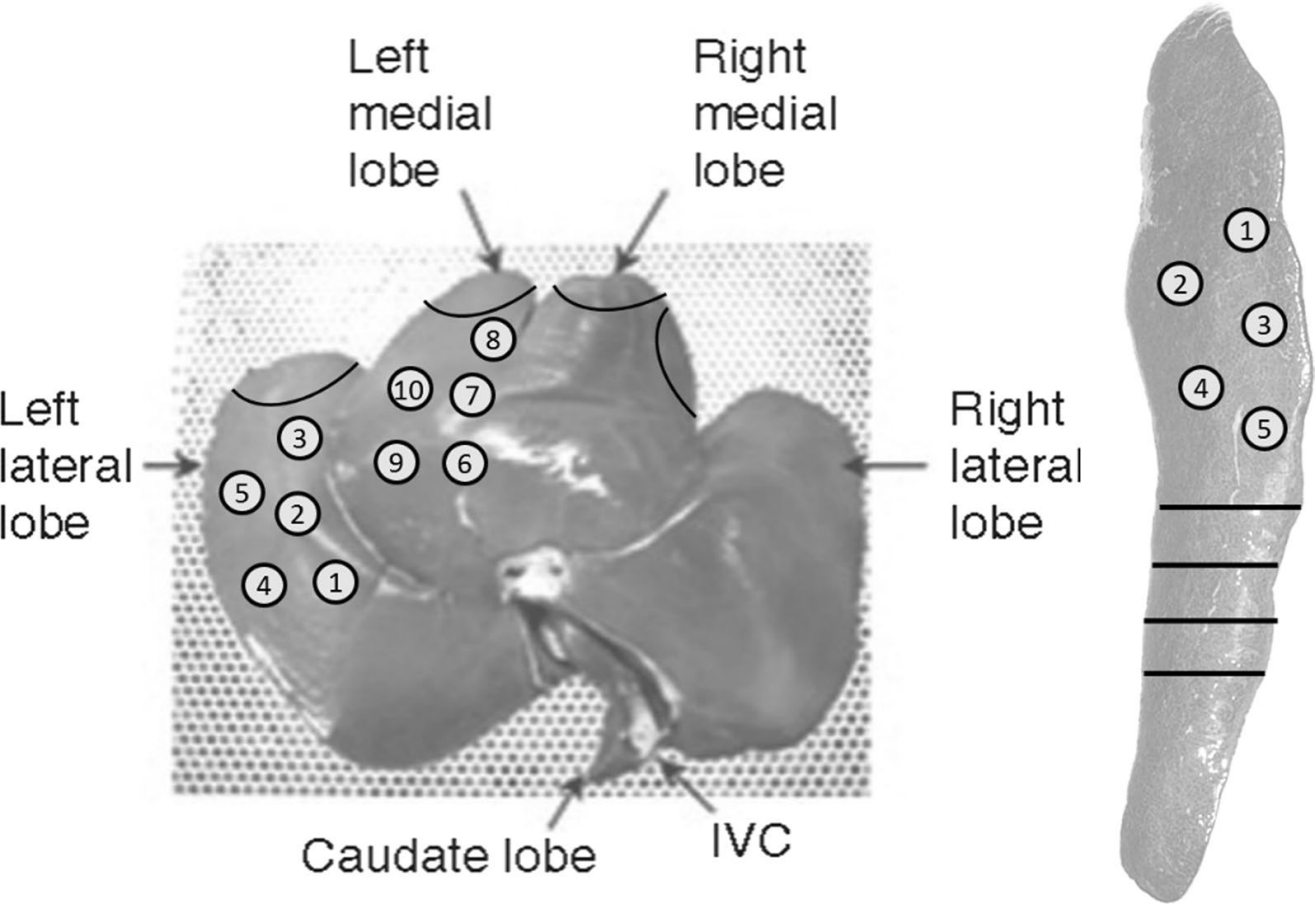
Locations of punch bleeding in experiment 1. (1–10, liver;1–5, spleen) and of resections in experiment 2 (black lines, liver at left; spleen at right. Black lines at spleen represent repeated resections

The study was divided into three experiments (Fig. 1). In experiment 1, twenty pigs underwent a laparotomy and were randomly divided into five groups of four animals surviving 1, 2, 3, 4 or 6 weeks. Each pig received 15 standardized punch bleedings, ten on the liver and five on the spleen. Five products (two prototype patches, two positive controls and one negative control) were applied three times (two on liver, one on spleen) per pig for evaluation of hemostasis of standardized moderate bleedings [23]. At relaparotomy after 1, 2, 3, 4 or 6 weeks, the sites of the former patch application were inspected for signs of tissue and foreign body responses and biopsied for histological analyses. After this inspection, pigs were used for a second experiment. During the relaparotomy, repeated partial liver lobe and spleen resections were performed on previously uninjured parts under normal and heparinized conditions (Fig. 2). The severe bleedings were treated with five products (three prototypes and two positive controls), and hemostasis was assessed.

Based on results of both experiments, the prototype GFC-NHS-POx patch was optimized by adding a cross-linker to reduce stickiness to surgical gauze (‘GATT-Patch’) and evaluated in a third experiment. In this experiment, eight pigs underwent a laparotomy to inflict standardized liver punch bleedings and perform partial liver and spleen resections for assessment of rapid, within 10s, hemostasis with the GATT-Patch and two positive and one negative controls.

### Surgical procedures

Pigs were anesthetized according to the standard protocol of the animal research facility (see appendix). After disinfection of the abdomen, sterile drapes were placed and a midline incision was performed from xyphoid to the umbilicus. During all surgeries, blood pressure, heart rate, CO_2_, O_2_ saturation, temperature and breathing frequencies were recorded after every five applications of patches. At the end of the surgery in experiment 1, the abdominal wall was closed in two layers: the fascia with a continuous suture (PDS VIO LOOP 150 cm M4, Ethicon, Sommerville, NJ) and the skin with intracutaneous continuous suture (Vicryl 2.0 Ethicon, Sommerville, NJ), after which anesthesia was ended, and the pig decannulated and brought to the pen to recover. At the end of the surgeries in experiment 2 and 3, animals were euthanized by an overdose of pentobarbital.

For the heparinized condition, heparin was given by intravenous bolus injection to increase activated clotting time (ACT) to 2–4 times the baseline value. ACT was measured with an Istat^®^ portable clinical analyzer (Abbott Point of Care Inc. Princeton, NJ USA) with ACT cartridges before the start of surgery for baseline, at least 5 min after heparin was administered and after each block of 5 treatments. Heparin was titrated according to ACT values to stay between 2 and 4 times baseline.

### Experiment 1: surgical punch (moderate) bleedings

Bleeding was inflicted with an 8 mm biopsy punch with a depth of 3 mm: *n* = 5 on the left lateral lobe of the liver, *n* = 5 on the left medial lobe of the liver and *n* = 5 on the spleen (Fig. 2). Blood flow was determined by collecting outflowing blood in a pre-weighed gauze for 30 s, measuring the difference in weight, corresponding to the amount of blood loss.

Each of three locations received five different patches; patches were allocated to the lesions using computer generated block randomization. Each patch was applied with digital pressure for one minute, except for TachoSil^®^, which needs a pressing time of three minutes according to the instructions for use [18]. Pressure was gently applied using a standard woven, wetted surgical gauze. Upon removal of the gauze, hemostasis was assessed by visual inspection by two observers at 10s (secondary efficacy endpoint) and 3 min (primary efficacy endpoint) after application. When the patch failed to stop the bleeding within 3 min after gauze removal, rescue measures (e.g., additional digital pressure) were taken to stop the bleeding with as final rescue measure the application of Veriset™.

At 1, 2, 3, 4 or 6 weeks, pigs underwent a relaparotomy, at which the abdominal cavity was macroscopically inspected for abnormalities. Adhesions to the liver and spleen at patch locations were categorized as: 0: no adhesions; 1: filmy adhesions, easy to separate by blunt dissection; 2: stronger adhesions, blunt dissection possible but partly sharp dissection necessary (beginning of vascularization); 3: strong adhesions, lysis possible but sharp dissection only, clear vascularization; and 4: very strong adhesions, lysis possible by sharp dissection only (organ strongly attached with severe adhesions and damage of organs hardly preventable) [24]. Macroscopic signs of patch remnants were evaluated as present or absent. Liver and spleen were biopsied at patch locations (10 × 5 mm and 5 mm deep) and stored in formaldehyde solution for histological analyses.

### Experiment 2: liver and spleen resection (severe) bleedings with and without heparin

At relaparotomy (experiment 1), after macroscopic inspection of the abdominal cavity, adhesion scoring and evaluation of patches’ remnants, the 20 pigs were reused for the second experiment. A series of four partial liver resections of 5 cm in length and 1.5 cm in depth was performed with surgical scissors (Fig. 2 provides an overview of the location of the resections) to generate a severe bleeding. The patch was folded around the resected area and digitally pressed with a wet gauze for two minutes (3 min for TachoSil^®^ per instructions for use). Upon removal of the gauze, hemostasis was assessed at 10s (secondary endpoint) and 3 min (primary endpoint), with rescue measures as described previously. Subsequently, four partial spleen resections ensuring the splenic artery was cut were made with surgical scissors to create an arterial, pulsating, severe bleeding. The patch was applied by folding it around the resected area and pressed as described for the liver resection. Upon removal of the gauze, hemostasis was again assessed at 10s and 3 min with rescue measures as previously described. After hemostasis, a new resection and bleeding was created ~ 3 cm more proximally on the spleen.

During experiment 2, each product was tested on severe bleedings four times on the liver and four times on spleen. These were distributed over two non-heparinized and two heparinized pigs.

### Experiment 3: punch bleedings and resections with short application time

After opening the abdomen and surgical exposure, liver punch bleedings and liver and spleen resections were performed followed by patch application, as described above, in eight pigs. Four patches’ types were evaluated, each applied on 6 liver punches, 6 liver resections and 2 spleen resections divided over the eight pigs. In contrast to the former experiments, pressure was applied for only 10s for all patches, including TachoSil^®^. After removal of the gauze, hemostatic efficacy was assessed at 10s (primary endpoint) and at 3 min (secondary endpoint). When after 10s no hemostasis was achieved, an additional 30s of pressure time was administered and repeated as needed until the 3-min secondary endpoint.

### Histology

Liver and spleen biopsies (experiment 1) were fixated using a 4% formaldehyde solution. Tissues were infiltrated and embedded in paraffin, sectioned (4 µm) using a microtome, pasted on superfrost-plus slides (Thermo-Scientific, Waltham, MA, USA) and dried overnight in the stove at 56 °C. Three different stains were done: HE (hematoxylin and eosin), MSB (Martius scarlet blue) and SR (Sirius red staining). All slides were digitalized using a Panoramic p250 scanner (3DHISTECH, Budapest, Hungary). For scoring, the slides were randomly mixed (for sequence). Two researchers independently scored the thickness of the patch, inflammatory cells and wound healing using a digital scoring form. The thickness of the patch was digitally measured (in mm) using Pannoramic viewer (3DHISTECH, Budapest, Hungary). Degradation of the patches in time was initially quantitatively scored by the number of times the patch was still visible by researchers during the surgical inspection of the patches and subsequently qualitatively by the microscopic thickness of the patch on histology. Inflammatory cells in the patch, new tissue and in the liver under the wound as well as the amount of fibroblasts in the new tissue were scored using the adjusted Ehrlich and Hunt scale [25] (scoring: 0: no evidence; 1: occasional evidence; 2: light scattering; 3: abundant presence; and 4: confluent cells or fibers). Wound healing was scored by general wound healing, according to Shafer [26] (scoring: 1: very light healing; 2: moderate healing; 3:advanced healing; and 4: well organized). The scores of the two researchers were compared; differences in scoring were discussed until consensus was reached.

### Statistical analysis

Continuous variables are presented as mean (SD); discrete data are presented as frequencies. Data on the primary and secondary endpoints of hemostatic efficacy in experiment 1 are presented as percentages of hemostatic success (pass/fail) at three minutes and linear mixed models were used for comparing efficacy between groups with post hoc LSD test; we tested for the dependency of the bleeding rate as a modifying factor confirming no correlation between bleedings within subjects. Hemostatic efficacy during experiment 2 and 3 was descriptively evaluated, and percentages of hemostatic success (pass/fail) were depicted. Furthermore, histological outcomes were descriptive evaluated without further analyses. All analyses were performed using SPSS (version 25, IBM corporation, Armonk, NY), and the significance level was set at a *p* value of < 0.05.

## Results

### Experiment 1

Baseline characteristics and experimental conditions of the groups are shown in Table 1. There were no differences in mean blood pressure at the time of surgical injury and in the severity of the biopsy punch bleeding as measured by blood volume. All operations were uneventful in all pigs and all but two recovered without complications. One pig had to be treated with adrenaline and diuretics immediately after the first operation, and one had a wound infection that was treated with local and systemic antibiotics. Both animals recovered and were eligible for the second surgery.

**Table 1:** Characteristics of bleeding among treatment groups

	TachoSil^®^	Veriset™	ORC-PLGA-NHS-POx: NU-POx	GFC-NHS-POx	GFC Blank	GFC-NHS-POx 1.5
*Experiment 1*						
Liver biopsy (*n*)	40	40	40	40	40	N/A
Blood flow (ml/min)*	8.2 (3.5)	8.2 (3.2)	7.4 (2.0)	7.9 (2.4)	7.8 (2.5)	N/A
Mean arterial pressure (mm Hg)	55.5 (11.7)	54.0 (11.7)	52.6 (7.6)	54.3 (11.8)	53.9 (12.3)	N/A
Spleen biopsy (*n*)	20	20	20	20	20	N/A
Blood flow (ml/min)*	7.7 (1.7)	6.9 (1.6)	6.8 (1.1)	7.4 (1.8)	6.9 (1.0)	N/A
Mean arterial pressure (mm Hg)	53.8 (11.9)	53.4 (12.0)	52.8 (10.3)	51.2 (6.9)	54.8 (11.6)	N/A
*Experiment 2 Non-heparinized pigs*						
Liver resections (*n*)	7	8	6	7	N/A	8
Spleen resections (*n*)	8	6	8	7	N/A	7
Mean arterial pressure (mm Hg)	49.0 (3.0)	46.7 (7.1)	47.4 (5.7)	37.2 (11.5)	N/A	46.1 (6.4)
ACT baseline (*s*)	98	90	98	86	N/A	102
ACT during experiment (*s*)	98	90	89	87	N/A	98
*Experiment 2 Heparinized pigs*						
Liver resections (*n*)	5	6	5	5	N/A	7
Spleen resections (*n*)	8	4	8	6	N/A	6
Mean arterial pressure (mm Hg)	49.9 (13.4)	42.4 (6.1)	56.7 (6.5)	46.0 (9.0)	N/A	50.4 (7.5)
ACT baseline (*s*)	92	87	92	98	N/A	101
ACT during experiment (*s*)	222	315	208	194	N/A	235

*ACT* Activated clotting time, *SD* standard deviation

*Blood flow depicted in ml/min but measured in weight per 30s

**Fig. 3:**
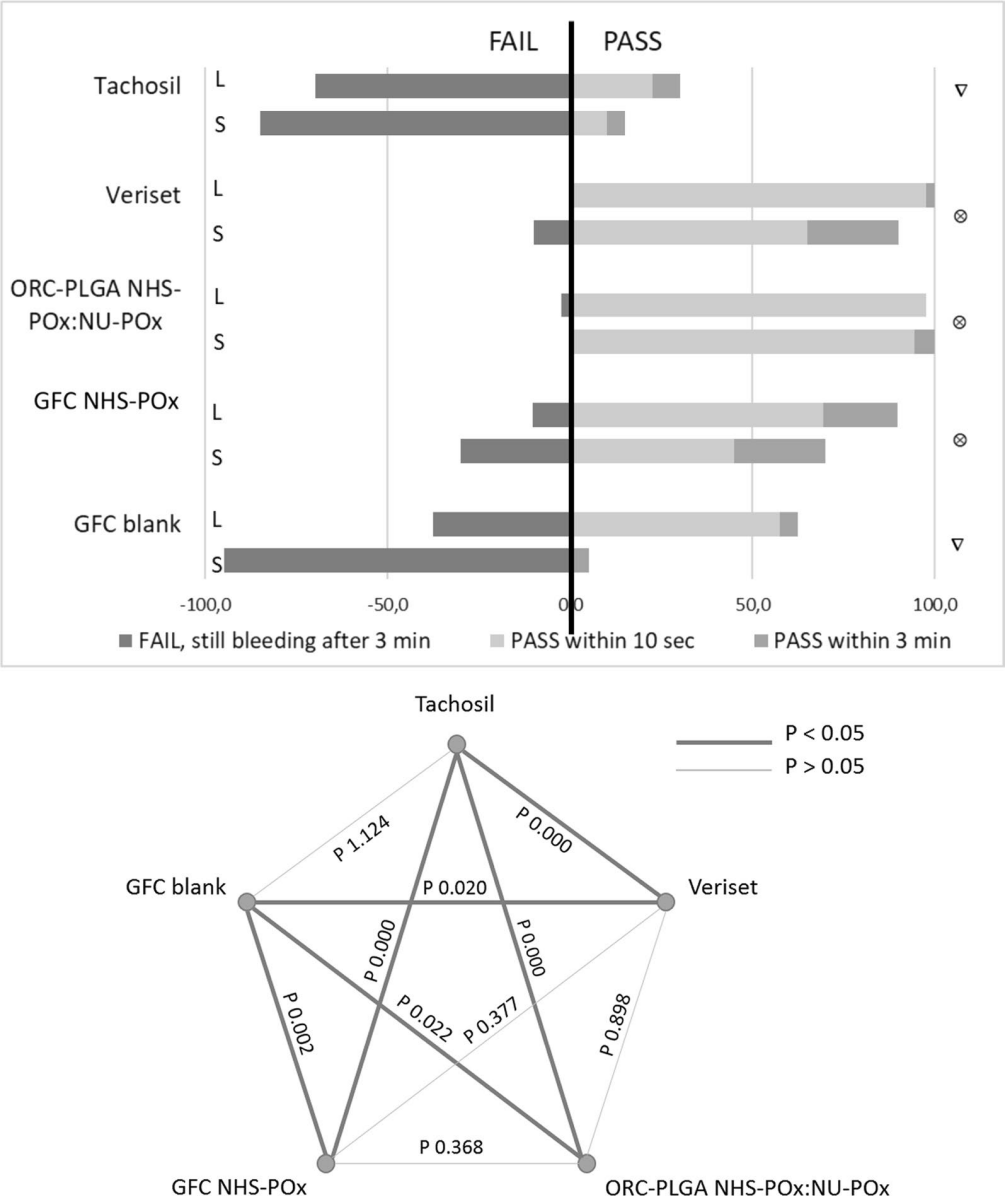
Efficacy of patches on standardized punch bleedings on liver (L) and spleen (S). $\otimes$ significantly different with ∇ (but not different to other $\otimes$); ∇ significantly different with $\otimes$ (but not different to other ∇)

GFC-NHS-POx (89.7% on liver, 70% on spleen; 83.1% combined), ORC-PLGA-NHS-POx: NU-POx (97.5% on liver, 100% on spleen; 98.3% combined) and Veriset™ (100% on liver, 90% on spleen; 96.7% combined) had better hemostatic efficacy compared to TachoSil^®^ (30.0% on liver, 15.0% on spleen; 25.0% combined) and GFC Blank (62.5% on liver, 5.0% on spleen; 43.3% combined) (Fig. 3). Hemostatic efficacy between GFC-NHS-POx, ORC-PLGA-NHS-POx: NU-POx and Veriset™ was comparable at 3 min. ORC-PLGA-NHS-POx: NU-POx resulted in less adherence to the tissue with a total of 13 patches (21.6%) coming loose after the 3-min observation, most frequently after manipulation for testing the following patch, which did not occur with any other patch that was evaluated.

**Fig. 4:**
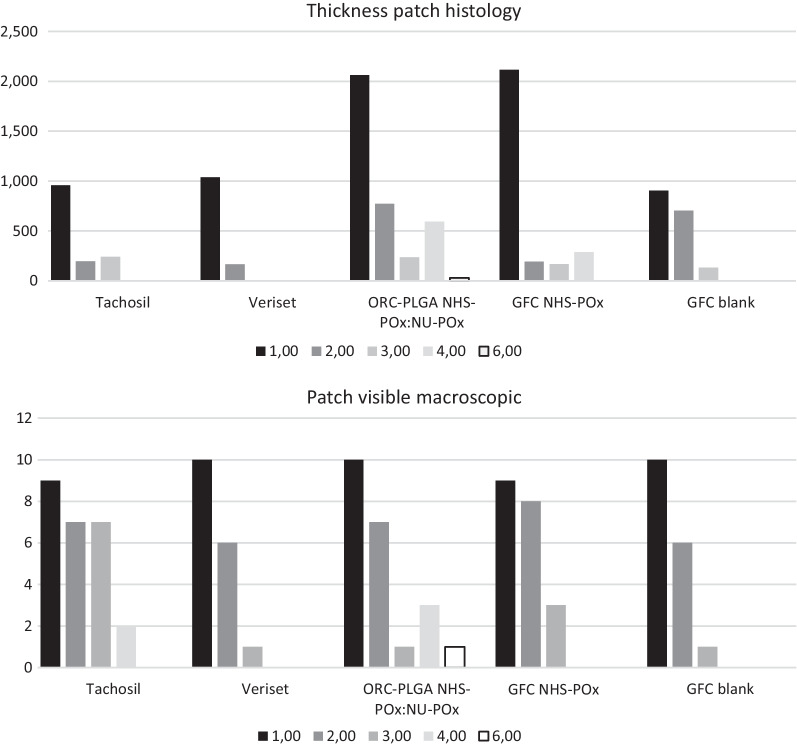
Degradation of patches in time (week 1–6) in terms of thickness of the patch measured histology (above) and the visibility macroscopically during inspections of the implanted patches (below)

**Fig. 5:**
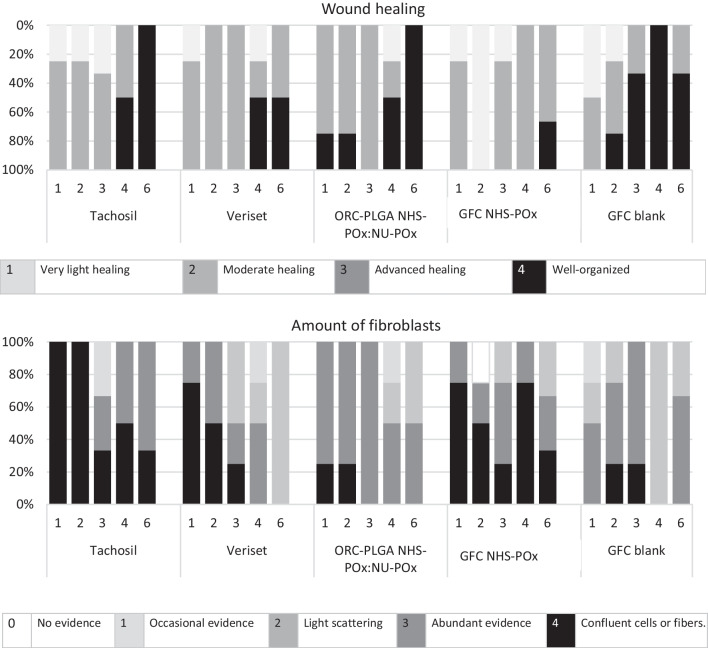
Wound healing according to Shafer (above) and the amount of fibroblast in the new tissue (below)

At relaparotomy for the inspection, adhesions were found in all animals and adhesiolysis was required; no difference in adhesion scoring between specific patches could be observed because adhesions covered a broad region and could not be adjudicated to a single patch. Visual inspection of the treatment sites demonstrated degradation of TachoSil^®^ within 3–4 weeks, Veriset™ within 2–3 weeks, ORC-PLGA-NHS-POx: NU-POx week 6 or longer, GFC-NHS-POx within 3–4 weeks and GFC Blank around week 3, as confirmed by histology (Fig. 4). Further histological analyses of wound healing demonstrated that wound healing generally progressed with the number of fibroblasts decreasing over time (Fig. 5). TachoSil^®^ showed more fibroblasts than Veriset™. Histological patterns of ORC-PLGA-NHS-POx: NU-POx and GFC Blank were comparable with Veriset™ and that of GFC-NHS-POx with TachoSil^®^.

### Experiment 2

At the time of severe bleeding creation, mean blood pressure showed some variation between the groups, with a markedly lower mean blood pressure in the GFC-NHS-POx group (37.2 mmHg) compared to between 46.1 and 49.0 mmHg in the other groups in the non-heparinized animals and a markedly higher mean blood pressure in the ORC-PLGA-NHS-POx: NU-POx group (65.7 mmHg) compared with between 42.4 and 50.4 mmHg in the other groups in the heparinized animals (Table 1). The ACT was comparable between treatment groups in the non-heparinized condition and showed some variation in the heparinized condition, although within target ranges.

**Fig. 6:**
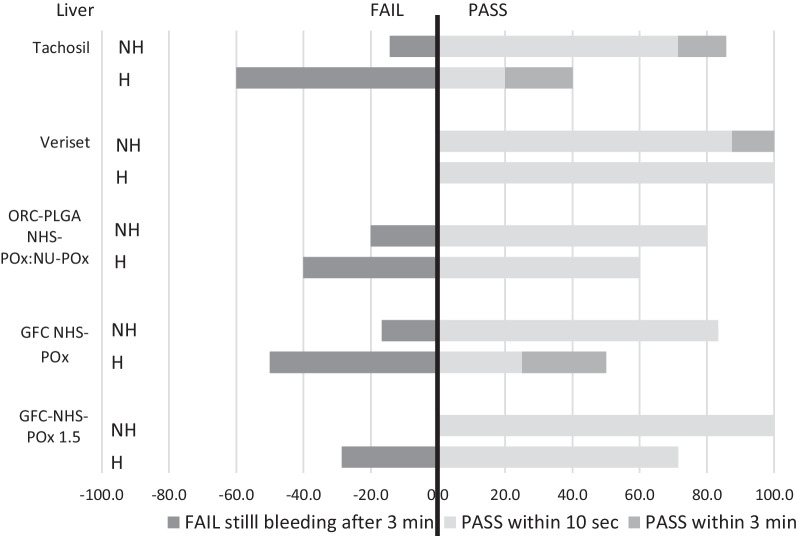
Efficacy of patches on partial liver resections in experiment 2 with (H) and without heparin (NH)

**Fig. 7:**
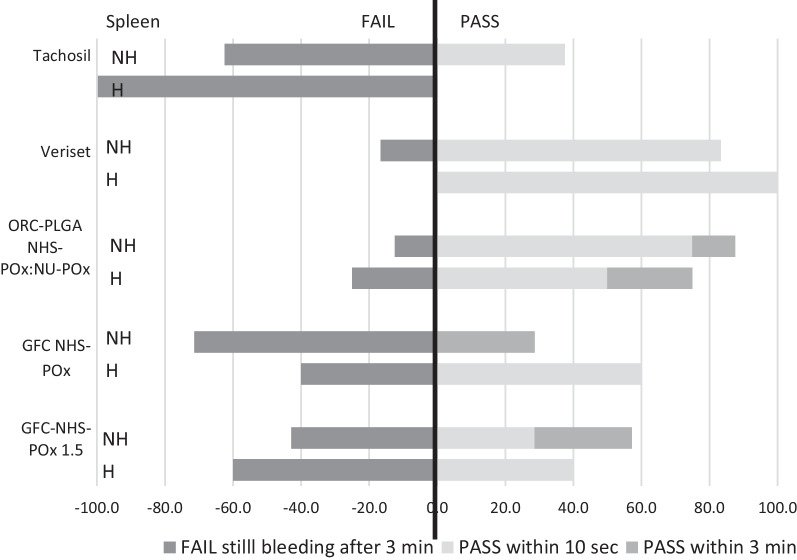
Efficacy of patches on partial spleen resections with severe pulsating arterial bleeding in experiment 2 with (H) and without heparin (NH)

Efficacy of the patches covering the liver defects is displayed in Fig. 6. Veriset™ had the highest percentage of success both (100%) with and without heparin, while TachoSil^®^ scored worst with heparin (40%). Success rates without heparin and with heparin were, respectively, 80% and 60% for ORC-PLGA-NHS-POx: NU-POx, 83.3% and 50% for GFC-NHS-POx and 100% and 71.4% for GFC-NHS-POx 1.5. Similar results were found regarding the spleen resections with arterial, pulsating severe bleedings (Fig. 7). Gauze removal after the application of both GFC-POx-based patches revealed adherence of the blood-drenched gauze to the patch, resulting in damage to the patch and (re)bleeding in all cases that did not achieve hemostasis in three minutes.

### Experiment 3

**Fig. 8:**
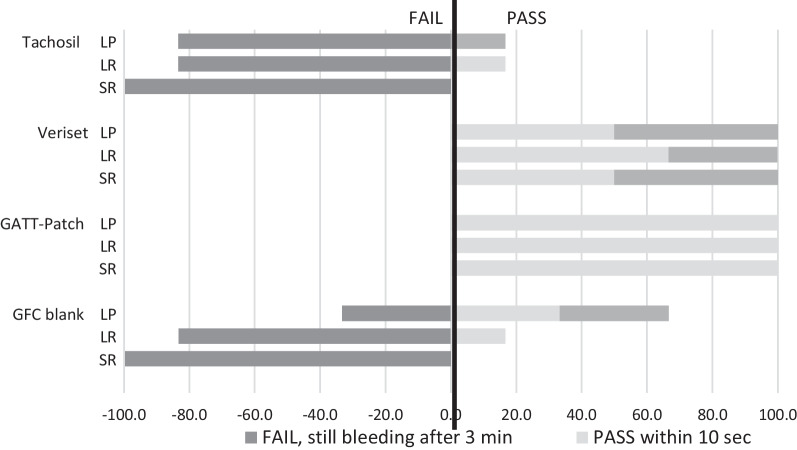
Efficacy of adjusted patch in experiment 3, LP liver punch, LR liver resection, SR spleen resection

The GATT-Patch (GFC-NHS-POx:NU-POx) reached hemostasis within 10s pressure of all punch and resection bleedings, with persistent hemostasis at three minutes (Fig. 8). Veriset™ application was successful in 50% of liver punch bleedings, 67% of liver resections and 50% of spleen resection within 10s and in all cases within three minutes. TachoSil^®^ and GFC Blank application were successful in only 0% and 33% of punch bleedings after 10s (17% and 67% in 3 min), 17% and 17% of liver resections after 10s (17% and 17% after 3 min) and 0% and 0% in spleen resections after 10s and after 3 min.

Considering all liver and spleen punctures and resections, GATT-Patch versus Veriset™, TachoSil^®^ and GFC Blank reached hemostasis in 10s in 100%, 42.8%, 7.1% and 14.3%, respectively, and within 3 min in 100%, 100%, 14.3% and 35.7%, respectively.

## Discussion

NHS-POx-based hemostatic patches demonstrate similar or better hemostatic efficacy as compared with common commercial surgical hemostatic patches in moderate bleedings of abdominal parenchymal organs in a surgically relevant porcine injury model. Hemostatic success seemed slightly less after partial liver and spleen resection, particularly after heparinization and compared to the best performing active control patch. However, a challenge test for short application and pressure time of 10s demonstrated superior hemostatic efficacy for the optimized GFC-NHS-POx:NU-POx patch to the best performing control patch. All experimental and control patches gradually degraded within six weeks, with signs of progressive wound healing and without related adverse events. Rapid and effective hemostasis combined with good surgical handling characteristics of this biodegradable ‘GATT-Patch’ holds promise for clinical application in liver and spleen bleedings under normal and compromised coagulant conditions.

Some of the prototype patches unexpectedly failed in severe bleedings, causing success differences between NHS-POx-based patches. The failure seemed to be caused by blood drenching the gauze through the patch. Blood penetrated the patch and accumulated in the gauze used for pressure, possibly reacted with the polymer inside the patch, causing adherence and rebleeding upon gauze removal. The GFC-NHS-POx better adhered to the organ surface and did not demonstrate this problem. The GFC-NHS-POx was therefore taken for further development. This resulted in a hemostatic patch that not only strongly adhered to parenchymal organ surfaces but outperformed both commercial patches regarding speed in hemostatic efficacy. Rapid hemostasis is much desired by surgeons to facilitate safe continuation of an operation and minimizing blood loss, especially in emergency situations.

A fair number of patches have been approved as an adjunct to hemostasis, but often require a long application time or are not effective on more severe bleedings [18]. TachoSil^®^ is frequently being referred to as the standard of care in challenging bleedings. More recent synthetic hemostatic NHS-PEG-based patches, such as Veriset™, are reportedly superior to TachoSil^®^ [17], supporting the choice of active control patches in our experiments. TachoSil^®^ failed in about 75 percent of moderate bleedings, which does not correspond with clinical experiences [27]. This may be due to the strict definition of hemostatic efficacy after three minutes with gauze pressure. Clinically a surgeon would continue pressure beyond this time until complete hemostasis, corresponding with the instructions for use. While in the current study hemostatic success may have been underestimated, others found comparable moderate efficacy, particularly after heparinization [28].

Although Veriset™ had excellent hemostatic properties in moderate and severe parenchymal bleedings, it has suboptimal handling characteristics compared to the best performing GFC-NHS-POx patch. Veriset™ is more rigid and is challenging to be folded onto irregular surfaces or applied into deep lesions. GFC-NHS-POx patches were specifically designed for flexibility to adapt to irregular tissue surfaces and to be folded around resection planes. Assessment of pliability was not part of the study design, and the results do not allow to draw firm conclusions regarding efficacy into a deep or irregularly shaped lesion, such as after excising a liver metastasis. Further advantage of GFC-NHS-POx is the complete impregnation of the patch with active ingredient on both sides, limiting application errors and allowing for stacking patches on top of each other for severe bleedings or to place overlapping patches over large bleeding surfaces.

Histological results up till six weeks of NHS-POx-based patches were comparable with control patches and point into the direction of clinical safety. Degradation of the experimental patch showed a linear pattern, corresponding with our and previous findings in commercial control patches [29, 30]. Although active GFC-NHS-POx patch seemed to degrade more slowly compared to the GFC Blank, it was complete within 4–6 week. This time is generally considered to be appropriate for complete wound healing without unnecessarily prolonged patch (foreign body) elicited inflammation [30]. We assessed adhesion formation to the liver as a safety measure for patch (degradation)-related inflammation and found no apparent differences between experimental and control patches. This finding should be interpreted with caution because patches were placed in close proximity at the liver injuries, hindering accurately allocating adhesions to a single patch. The almost 100 percent of animals with adhesions at the patch localizations at all time points could be considered a serious related adverse event. However, we cannot rule out that adhesions were due to the surgical procedure and bleeding from injuries, as is expected with major abdominal surgery irrespective of applying hemostatic patches [31, 32].

We consider the translational value of results as high, using a large animal that resembles human anatomy, physiology, blood flow and tissue properties [33, 34], and surgical injuries with bleedings that are eligible for use of hemostatic patches in patients. The evaluation of patch performance under anticoagulant conditions and in severe bleeding add to this value, considering that an increasing number of patients with anticoagulant therapy will undergo increasingly complex parenchymal organ surgery [35, 36].

The GFC-NHS-POX:NU-POx (GATT-Patch) was further studied for regulatory purposes with confirmation of hemostatic efficacy, minimal local tissue reaction, complete degradation within 28 days of implantation and comparable postoperative adhesion formation to existing hemostatic products (unpublished data). Currently, GATT-Patch is being evaluated in a single arm, first-in-human study of open liver surgery (Clinicaltrials.gov identifier NCT04819945) for clinical safety and efficacy.

## Conclusions

NHS-POx-based patches, particularly the latest iteration, GATT-Patch, are fast in achieving effective hemostatic sealing on standardized moderate and severe bleedings on liver and spleen as compared with currently available hemostatic patches. As they appear safe during follow-up, it offers a promising alternative to currently approved hemostatic and/or sealant patches for use in a wide variety of elective and emergency abdominal operations.

## Data Availability

The dataset generated during the current study is available from the corresponding author on reasonable request.

## Appendix

### Anaesthesia protocol

**Table:**

Time	Agent	Dosage		Route of administration	Name
*Preparatio*n					
-24 hr	Meloxicam	0.6	mg/kg	Per Os	Novem ®
-15 min	Ketamine	10	mg/kg	Intramuscular	Ketamine
-15 min	Midazolam	1	mg/kg	Intramuscular	Dormicum®
-15 min	Atropine	50	µg/kg	Intramuscular	Atropinesulfate
-5 min	Amoxicillin	20	mg/kg	Intravenous	Amoxicillin
*Induction*					
0 min	Propofol	2.50	mg/kg	Intravenous	Propofol
*Maintenance *					
1 min	Isoflurane	0.5	%ET	Per Os	Isoflurane FiO_2_ 35%
3 min	Midazolam	0.6	mg/kg/hr	Intravenous	Dormicum®
4 min	Sufentanil	5	µg/kg	Intravenous	Sufenta Forte
5 min	Sufentanil	10	µg/kg/hr	Intravenous	Sufenta Forte
5 min	Meloxicam	0.5	mg/kg	Intramuscular	Novem ®
60 min	Atropine	25	µg/kg	Intramuscular	Atropinesulfate
60 min	Rocuronium	0.2	mg/kg	Intravenous	Rocuroniumbromide
60 min	Rocuronium	0.4	mg/kg/hr	Intravenous	Rocuroniumbromide
*Recovery*					
-45 min	Sufentanil	0	µg/kg/hr	Intravenous	Sufenta Forte
-45 min	Buprenorphin	10	µg/kg	Intravenous	Bupaq
*Postoperative*					
24 hr	Meloxicam	0.6	mg/kg	Per Os	Metacam
12 en 24 hr	Buprenorfin	10	u/kg	Intramuscular	Bupaq

## Abbreviations

NHS-Pox: *N*-Hydroxy-succinimide polyoxazoline
GFC: Gelatin fibrous carrier
ORC: Oxidized regenerated cellulose
NU-POx: Nucleophilically activated polyoxazoline
PLGA: Poly(lactic-co-glycolic acid)
ARRIVE: Animal research: reporting in vivo experiments
HE: Hematoxylin and eosin
MSB: Martius scarlet blue
SR: Sirius red
